# Predictors of Cyberchondria During the COVID-19 Pandemic: Cross-sectional Study Using Supervised Machine Learning

**DOI:** 10.2196/42206

**Published:** 2023-04-25

**Authors:** Alexandre Infanti, Vladan Starcevic, Adriano Schimmenti, Yasser Khazaal, Laurent Karila, Alessandro Giardina, Maèva Flayelle, Seyedeh Boshra Hedayatzadeh Razavi, Stéphanie Baggio, Claus Vögele, Joël Billieux

**Affiliations:** 1 Department of Behavioural and Cognitive Sciences University of Luxembourg Esch-sur-Alzette Luxembourg; 2 Department of Psychiatry Nepean Hospital Penrith Australia; 3 Discipline of Psychiatry Faculty of Medicine and Health Sydney Medical School Nepean Clinical School, University of Sydney Sydney Australia; 4 Faculty of Human and Social Sciences Kore University of Enna Enna Italy; 5 Addiction Medicine Department of Psychiatry Lausanne University Hospital Lausanne Switzerland; 6 Department of Psychiatry and Addictology University of Montreal Montreal, QC Canada; 7 Department of Psychiatry Lausanne University Lausanne Switzerland; 8 Centre d'Enseignement de Recherche et de Traitement des Addictions Hôpital Universitaire Paul Brousse, Université Paris-Saclay Villejuif France; 9 Institute of Psychology University of Lausanne Lausanne Switzerland; 10 Division of Prison Health Geneva University Hospitals and University of Geneva Thônex Switzerland; 11 Institute of Primary Health Care University of Bern Bern Switzerland

**Keywords:** cyberchondria, COVID-19, online health information, fear of COVID-19, health anxiety, machine learning

## Abstract

**Background:**

Cyberchondria is characterized by repeated and compulsive online searches for health information, resulting in increased health anxiety and distress. It has been conceptualized as a multidimensional construct fueled by both anxiety and compulsivity-related factors and described as a “transdiagnostic compulsive behavioral syndrome,” which is associated with health anxiety, problematic internet use, and obsessive-compulsive symptoms. Cyberchondria is not included in the *International Classification of Diseases 11th Revision* or the *Diagnostic and Statistical Manual of Mental Disorders, Fifth Edition*, and its defining features, etiological mechanisms, and assessment continue to be debated.

**Objective:**

This study aims to investigate changes in the severity of cyberchondria during the COVID-19 pandemic and identify the predictors of cyberchondria at this time.

**Methods:**

Data collection started on May 4, 2020, and ended on June 10, 2020, which corresponds to the first wave of the COVID-19 pandemic in Europe. At the time the study took place, French-speaking countries in Europe (France, Switzerland, Belgium, and Luxembourg) all implemented lockdown or semilockdown measures. The survey consisted of a questionnaire collecting demographic information (sex, age, education level, and country of residence) and information about socioeconomic circumstances during the first lockdown (eg, economic situation, housing, and employment status) and was followed by several instruments assessing various psychological and health-related constructs. Inclusion criteria for the study were being at least 18 years of age and having a good understanding of French. Self-report data were collected from 725 participants aged 18-77 (mean 33.29, SD 12.88) years, with females constituting the majority (416/725, 57.4%).

**Results:**

The results showed that the COVID-19 pandemic affected various facets of cyberchondria: cyberchondria-related distress and compulsion increased (distress *z*=–3.651, *P*<.001; compulsion *z*=–5.697, *P*<.001), whereas the reassurance facet of cyberchondria decreased (*z*=–6.680, *P*<.001). In addition, COVID-19–related fears and health anxiety emerged as the strongest predictors of cyberchondria-related distress and interference with functioning during the pandemic.

**Conclusions:**

These findings provide evidence of the impact of the COVID-19 pandemic on cyberchondria and identify factors that should be considered in efforts to prevent and manage cyberchondria at times of public health crises. In addition, they are consistent with a theoretical model of cyberchondria during the COVID-19 pandemic proposed in 2020. These findings have implications for the conceptualization and future assessment of cyberchondria.

## Introduction

### Background

The COVID-19 pandemic and related mitigation measures have drastically changed our lives. Although political efforts have somewhat alleviated the economic and public health consequences of the pandemic, experts have warned that its long-term effects on mental health tend to be neglected [1-3]. Research conducted since the initial outbreak of the COVID-19 pandemic in China showed an increase in general stress [4] and a substantial increase in psychopathological symptoms that are frequently encountered in clinically relevant mood or anxiety disorders or both [5,6]. Preliminary evidence also suggests that survivors of COVID-19 appear to be at increased risk for mental health problems [7].

Worries and fear are centrally involved in COVID-19–related psychopathologies and problematic behaviors [8-11]. Schimmenti and coworkers [12,13] proposed a model to account for fear experiences during the COVID-19 pandemic. This model posits that several domains of fear (bodily, relational/interpersonal, cognitive, and behavioral) interact and contribute to the onset and perpetuation of COVID-19–related psychological distress through maladaptive, repetitive, and functionally impairing behaviors. One such behavior used to gain control over fear during the COVID-19 pandemic concerns compulsive searches for online health information, or “cyberchondria” [12,14,15].

Cyberchondria is defined as a poorly controlled pattern of searching for health-related information online, resulting in heightened health anxiety and other negative consequences (eg, interference with work or relationships and psychological distress), which can be functionally impairing and are associated with abnormal healthcare use [16,17]. Cyberchondria has been conceptualized as a multidimensional construct fueled by both anxiety and compulsivity-related factors [18] and described as a “transdiagnostic compulsive behavioral syndrome” [19], which is associated with health anxiety, problematic internet use, and obsessive-compulsive symptoms [20,21]. Cyberchondria is not included in the *International Classification of Diseases 11th Revision* or the *Diagnostic and Statistical Manual of Mental Disorders, Fifth Edition*, and its defining features, etiological mechanisms, and assessment continue to be debated [22]. The upshot of this situation is that reliable data on the prevalence of cyberchondria in the general population are not available [19,23]. Nevertheless, preliminary data suggest that cyberchondria might be commonly encountered [24] and that it might be more frequent in patients with various medical conditions [25,26]. With regard to its psychological correlates, previous research has shown that cyberchondria is associated with low self-esteem, dysfunctional meta-cognitive beliefs, heightened anxiety sensitivity, and intolerance of uncertainty, as well as a tendency toward pain catastrophizing [19].

According to Starcevic et al [14], the COVID-19 context is likely to have contributed to the occurrence of cyberchondria or exacerbated it for several reasons: (1) there is a heightened perception of threat and the accompanying fear due to a recently identified and poorly understood disease; (2) uncertainty concerning the pandemic and the effectiveness of various mitigating measures (eg, lockdowns and vaccination) undermines attempts to cope with the situation; (3) the paucity of authoritative, trustworthy, and evidence-based health information further thwarts coping efforts; (4) the abundance of confusing, conflicting, unverified, and constantly updated information amplifies bewilderment; and (5) engaging in excessive online health information seeking cannot provide the necessary information and reassurance. These factors have been posited to increase fear and distress, thereby also increasing the perception of threat, further reducing effective coping with uncertainty and perpetuating online health searches. It is worth noting that the psychological model of cyberchondria during the COVID-19 described here [14] was developed at a time (March-May 2020) when the uncertainties surrounding the pandemic were at their maximum level and when the data for this research were collected.

In addition to this theoretical account, there is a growing number of empirical, mainly cross-sectional research reports focusing on various aspects of cyberchondria during the COVID-19 pandemic. Several important findings, in line with the psychological model proposed by Starcevic et al [14], have emerged from these studies. First, a strong relationship was found between cyberchondria and the fear of COVID-19 [27-30], with some studies reporting that cyberchondria predicts the fear of COVID-19 [29], other studies suggesting that the reverse might be true (ie, that the fear of COVID-19 predicts cyberchondria [30]), and yet other research reporting that both cyberchondria and health anxiety are risk factors for the fear of COVID-19 [27]. Second, several reports have confirmed the important role of intolerance of uncertainty during the pandemic, although the precise nature of its relationship with cyberchondria differs between studies [30-32]. Third, information overload was found to predict cyberchondria during the pandemic [33], whereas excessive and misleading information usually obtained through social media resulted in both cyberchondria and information overload [34]. Using a 2-wave longitudinal design during the initial outbreak of the pandemic in Europe, Jokic-Begic et al [35] showed that cyberchondria played a moderating role in the increase in the fear of COVID-19 between time 1 (when the first COVID-19 patients were diagnosed) and time 2 (when lockdown was introduced). Although these studies have improved our understanding of cyberchondria during the COVID-19 pandemic, much remains unknown about the psychological factors that contribute to the development of cyberchondria in the COVID-19 context.

### Aims of the Study

In line with the assumption that cyberchondria is an important public health issue in the COVID-19 context [14,15], the objectives of this study were 2-fold. First, we investigated the levels of cyberchondria during the pandemic and compared them with the retrospectively assessed prepandemic levels of cyberchondria. Second, we aimed to identify the psychological factors that predicted cyberchondria during the pandemic. The selection of predictor variables was based on the psychological model of cyberchondria during COVID-19 [14], including the intolerance of uncertainty, COVID-19–related fears, health anxiety, and somatic symptoms. At the time the study was designed and conducted, the psychological model of Starcevic et al [14] was not yet published. Yet, some of the authors of this study were involved in its development and were thus able to capitalize on it for the selection of variables to be included in this study. In addition, we assessed impulsivity traits and attachment styles as predictor variables, because these psychological dimensions are potentially of relevance for behavioral patterns such as cyberchondria, which are characterized by diminished control and interpersonal difficulties [19]. To build a robust predictive model, this study used supervised machine learning–based regression models (elastic net regression).

## Methods

### Procedure

Participants for this study were recruited using an online survey (created with *Qualtrics*), which was disseminated via social media (ie, Twitter, LinkedIn, Facebook, and Instagram). The study was also disseminated via the research networks of the authors and the scientific societies they are affiliated with. Data collection started on May 4, 2020, and ended on June 10, 2020, which corresponds to the first wave of the COVID-19 pandemic in Europe. At the time this study took place, French-speaking countries in Europe (France, Switzerland, Belgium, and Luxembourg) all implemented lockdown or semilockdown measures. The survey consisted of a questionnaire collecting demographic information (sex, age, education level, and country of residence) and information about socioeconomic circumstances during the first lockdown (eg, economic situation, housing, and employment status) and was followed by several instruments assessing various psychological and health-related constructs. The entire survey was administered in French. The survey software was set up in a way that participants could not skip any question, and therefore, we had no missing or incomplete responses in the final data set.

Some of the independent Italian data related to this project have been published elsewhere [10]. A list of all measures used in the online survey (including measures not considered here) is available from the Open Science Framework (OSF) [36]. All data, codes, and materials are available from the OSF link provided [36].

### Participants

Inclusion criteria for the study were being at least 18 years of age and having a good understanding of French. No specific exclusion criteria were used. Sociodemographic characteristics of the participants are reported in Table 1. The sample consisted of 725 participants aged 18-77 (mean 33.29, SD 12.88) years, with females constituting the majority (416/725, 57.4%). Regarding a pandemic-related living situation, 5% (36/725) reported living with roommates during the lockdown, 20.4% (148/725) lived alone, 26.8% (194/725) lived with their children, 27.6% (200/725) lived with their parents, and 43.3% (314/725) lived as a couple. Most of the sample (626/725, 86.3%) assessed their housing situation as adequate during the lockdown. With regard to their financial situation, the majority of the sample (451/725, 62.2%) reported that they experienced no changes during the lockdown.

**Table 1:** Sociodemographic characteristics of the study sample (N=725).

Characteristics		Participants, n (%)
**Gender**		
	Male	302 (41.7)
	Female	416 (57.4)
	Nonbinary	7 (1.0)
**Education**		
	Lower secondary	23 (3.2)
	Upper secondary	102 (14.1)
	Bachelor’s degree	308 (42.5)
	Master’s degree	236 (32.6)
	Doctoral degree	56 (7.7)
**Profession**		
	Employed	385 (53.1)
	Unemployed	64 (8.8)
	Retired	16 (2.2)
	Full-time student	223 (30.8)
	Other	37 (5.1)
**Country of residence**		
	Switzerland	64 (8.8)
	France	479 (66.1)
	Belgium	45 (6.2)
	Other	137 (18.9)
**Living situation**		
	Live with flat mate(s)	36 (5.0)
	Live alone	148 (20.4)
	Live with children	194 (26.8)
	Live with parents	200 (27.6)
	Live with partner	314 (43.3)
	Other	87 (12.0)
**Quality of housing situation during the pandemic**		
	Adequate	626 (86.3)
	Inadequate	99 (13.7)
**Economic situation during the pandemic**		
	Worse than before	194 (26.8)
	No changes	451 (62.2)
	Better than before	80 (11.0)

### Ethical Considerations

Participation was anonymous and voluntary. No compensation for completing the survey was provided. Participants were informed about the aims of the survey before they signed electronic informed consent. The study received approval from the Institutional Review Board for psychological research of the Kore University of Enna (UKE), in the framework of a joint Italian and Swiss research program on cyberchondria and COVID-19–related fears (code: UKE-IRBPSY-04.20.04).

### Measures

#### Cyberchondria Severity Scale – Short Form

The Cyberchondria Severity Scale – Short Form (CSS-12) [37] is a short 12-item version of the original 33-item CSS [18], which assesses the severity of cyberchondria. Items are rated on a 5-point Likert scale from 1 (never) to 5 (always). The global severity of cyberchondria is reported by using the total score derived from the 12 items. The psychometric properties of the CSS-12 have been reported by previous studies, and its factor structure has been established by a combination of exploratory and confirmatory factor analyses [37,38]. The CSS-12 was shown to measure 4 different dimensions of cyberchondria: *excessiveness* (eg, “I enter the same symptoms into a web search on more than 1 occasion”), *distress* (eg, “I feel more anxious or distressed after researching symptoms or perceived medical conditions online”), *reassurance* (eg, “Researching symptoms or perceived medical conditions online leads me to consult with my general practitioner”), and *compulsion* (eg, “Researching symptoms or perceived medical conditions online interrupts my offline social activities”). In this study, participants were asked to provide 2 different responses for each CSS-12 item: one response was related to a general or “normal” context (ie, before the COVID-19 pandemic), while the other was related specifically to the COVID-19 context. As we adapted the response format without changing any item wording, we verified separately the factorial structure of the data obtained from each response format. Confirmatory factor analyses showed that the previously established 4-factor structure (excessiveness, distress, reassurance, and compulsion) fitted well our data obtained from both response formats (ie, “before COVID-19” and “during COVID-19”). Confirmatory factor analyses conducted on our adapted CSS-12 are available from the OSF [36].

#### Multidimensional Assessment of COVID-19–Related Fears

The Multidimensional Assessment of COVID-19–Related Fears (MAC-RF) [10] consists of 8 items that assess various domains of COVID-19–related fears. Items are rated on a 5-point Likert scale from 0 (very unlike me) to 4 (very like me). The fear domains assessed include the bodily domain (fear for the body and fear of the body, eg, “I am frightened about my body being in contact with objects contaminated by the coronavirus”), the interpersonal domain (fear for significant others and fear of significant others, eg, “I am frightened about my family members or close friends being in contact with other people and becoming infected with the coronavirus”), the cognitive domain (fear of knowing and fear of not knowing, eg, “I do not want to be exposed to information about the coronavirus infection, because it makes me feel upset and anxious”), and the behavioral domain (fear of taking action and fear of inaction, eg, “During the coronavirus pandemic, I feel paralyzed by indecisiveness or the fear of doing something wrong”). The psychometric properties of the scale have been established via item-response theory and relationships with convergent psychological constructs [10]. In this study, a total score of COVID-19–related fears was used.

#### Intolerance of Uncertainty Scale – Short Form

The Intolerance of Uncertainty Scale – Short Form (IUS-12) [39] is a 12-item version of the original 27-item IUS [40], which measures the intolerance of uncertainty. Items are rated on a 5-point Likert scale from 1 (not representative at all) to 5 (completely representative). Higher scores signal higher intolerance of uncertainty. The scale provides a total score and scores on 2 dimensions of intolerance of uncertainty: inhibitory (eg, “When I am uncertain, I cannot function very well”) and prospective (eg, “It frustrates me not having all the information I need”). Following the approach of a previous study relating the intolerance of uncertainty to cyberchondria [41] and the recommendation by Carleton et al [39], a total score on the IUS-12 was used to evaluate intolerance of uncertainty.

#### The 15-Item Patient Health Questionnaire

The 15-Item Patient Health Questionnaire (PHQ-15) [42] measures the severity of common somatic symptoms (abdominal pain, headache, nausea, and others) experienced during the previous month. The PHQ-15 is often used as a measure of somatic symptom proneness (eg, Ref. [43]), and it has been shown to be useful in identifying somatic symptom disorder [44]. Each item assesses the degree to which individuals experience a specific somatic symptom rated on a scale from 0 (not bothered at all) to 2 (bothered a lot), with higher scores indicating a greater severity of somatic symptoms. One item pertains to menstrual pain, but this item was kept for the entire sample to ensure that male transgender participants could rate this item, when appropriate. Scores on the PHQ-15 correlated with the severity of disability and functional impairment related to somatic problems [42].

#### Short Health Anxiety Inventory

The Short Health Anxiety Inventory (SHAI) is a short form version of the original 64-item HAI [45,46]. The questionnaire is composed of 18 items that evaluate the degree of individuals’ worries about their own health adapted for non-treatment-seeking individuals. Each item is scored between 0 to 3, depending on the response provided (eg, item 1 is rated as follows: 0=“I do not worry about my health”; 1=“I occasionally worry about my health”; 2=“I spend much of my time worrying about my health”; and 3=“I spend most of my time worrying about my health”). Scores range between 0 and 54, with higher scores indicating a greater severity of health anxiety. The SHAI demonstrated good convergent and discriminant validity [45]. In this study, the total score of the measure was used.

#### Relationship Questionnaires

The Relationship Questionnaire (RQ) [47] is a 4-item scale investigating 4 prototypical adult attachment styles: secure, dismissing, preoccupied, and fearful. Each attachment style is evaluated through a first-person statement. Participants are asked to evaluate the correspondence of each statement with their relationship attitudes on a 7-point Likert scale from 1 (strongly disagree) to 7 (strongly agree). An example of an item (dismissing style) is “I am comfortable without close emotional relationships. It is very important to me to feel independent and self-sufficient, and I prefer not to depend on others or have others depend on me.”

The RQ has been shown to possess good test-retest reliability and discriminant validity [48,49] and has been successfully used in research focusing on internet-mediated problematic behaviors [50].

#### Short UPPS-P Impulsive Behavior Scale

The Short UPPS-P Impulsive Behavior Scale (s-UPPS-P) [51] is a short 20-item version of the original 59-item UPPS-P Impulsive Behavior Scale [52,53]. Items are rated on a 4-point Likert scale from 1 (I agree strongly) to 5 (I disagree strongly). The s-UPPS-P measures 5 different impulsivity dimensions (4 items per dimension), namely negative urgency (eg, “When I am upset, I often act without thinking”), positive urgency (eg, “When I am really excited, I tend not to think on the consequences of my actions”), lack of premeditation (eg, “Before making up my mind, I consider all the advantages and disadvantages”—reverse-scored item), lack of perseverance (eg, “I finish what I start”—reverse-scored item), and sensation seeking (eg, “Sometimes, I like doing things that are a bit frightening”). The psychometric properties of the s-UPPS-P (eg, factor structure, item-based network structure, test-retest reliability, association with convergent constructs) have been established in previous studies [51,54]. In this study, a global score of “general urgency” was used, as recent research shows that positive and negative urgency form a single coherent construct [54].

### Statistical Analysis

Our first aim was to test whether the levels of cyberchondria increased during the pandemic in comparison with a retrospectively assessed cyberchondria, based on the CSS-12. As the CSS-12 scores in both response formats did not follow a normal distribution, we relied on nonparametric tests and computed Wilcoxon signed-rank tests for dependent samples. We also reported on the effects of gender, age, and education on the CSS-12 scores during COVID-19. The effect of gender was tested using the Mann-Whitney U test (nonbinary participants were not considered in this analysis due to their low number), and the effects of age and education were tested using Kruskal-Wallis tests (see Table 2 for more details).

**Table 2:** CSS-12^a^ scores before and during COVID-19.

Scores	Score before COVID-19, mean (SD)	Score during COVID-19, mean (SD)	Score before COVID-19, median	Score during COVID-19, median	*z*	P value	Effect size
Total CSS-12 scores	26.68 (8.04)	26.64 (8.88)	26	26	–0.150	.88	0.006
CSS-12 excessiveness subscale scores	9.36 (2.85)	9.26 (3.06)	9	9	–0.763	.45	0.028
CSS-12 distress subscale scores	6.67 (2.88)	6.83 (3.12)	6	6	–3.651	<.001	0.136
CSS-12 reassurance subscale scores	5.90 (2.32)	5.54 (2.48)	6	5	–6.680	<.001	0.248
CSS-12 compulsion subscale scores	4.75 (2.24)	5.00 (2.51)	5	4	–5.697	<.001	0.212

^a^CSS-12: Cyberchondria Severity Scale – Short Form.

Our second aim was to determine the factors that predicted cyberchondria during the pandemic, based on the psychological model elaborated by Starcevic et al [14]. Our predictive models focused on the CSS-12 subscales, which were most impacted by the COVID-19 pandemic (ie, those whose scores differed significantly from before the pandemic). Potential predictors for each model computed were selected based on their correlations with the dependent variable (ie, the CSS-12 subscales most impacted by the pandemic). Because we planned to apply a regression model, we did use Spearman correlations to select our predictors. Indeed, correlations can be used to quantify the dependence between our potential predictors and our dependent variable. Thus, all candidate predictor variables whose correlations with the dependent variable were ≥0.30 (which corresponds to a moderate effect size [55,56]) were retained and included in our predictive models. A series of predictive regression models were then computed based on a supervised machine learning approach.

Supervised machine learning approaches are generally defined as “a set of methods that can automatically detect patterns in data, and then use the uncovered patterns to predict future data” [57]. Traditional multiple linear regression models are limited in the sense that they rely on the entire sample to fit a model and test their accuracy. These models are also susceptible to bias and may be “overoptimistic” in terms of the variance explained or generalization to other independent samples. In contrast, the basic principle of the supervised machine learning approach is to shuffle the data (using a “seed,” which is a value set as a reference point to generate the randomization of the data) and then split them into 2 independent subsamples: one subsample is used to fit the model (train set, 60%-80% of the data), while the other is used to test the model’s accuracy (test set, 20%-40% of the data). Compared to the traditional regression approach, this method is generally considered to be more reliable and to produce more robust findings as the accuracy of the computed predictive model is derived from a new and independent sample with unknown variance [58,59]. Yet, such an approach needs a large sample to produce reliable findings, and another data-splitting strategy has been proposed in the context of supervised machine learning if the sample size is limited. This strategy is called cross-validation and involves a series of runs whereby the entire data set is split into several folds, which are all used as train and test sets [60]. In each run, a unique fold is used to determine the accuracy of the model computed, while the other folds are used to fit the model. Finally, each fold is used as a test set in one run and as a part of the train set in the other runs. The cross-validated score is obtained by computing a mean accuracy score based on the runs launched. This method is often used within the train set to “tune” the hyperparameters (a value that can be specified by the researcher) of a machine learning model. The fold used to compute cross-validation accuracy is called the validation set. Tuning a model consists of finding the hyperparameters that produce the best possible score on the validation set. When the hyperparameters are identified, the model is then refitted on the entire train set and its accuracy is evaluated using the test set. Nevertheless, this method has been criticized for promoting “overfitting,” in the sense that the model and its hyperparameters are too specific to the train set, thus potentially limiting its reproducibility [59].

**Figure 1:**
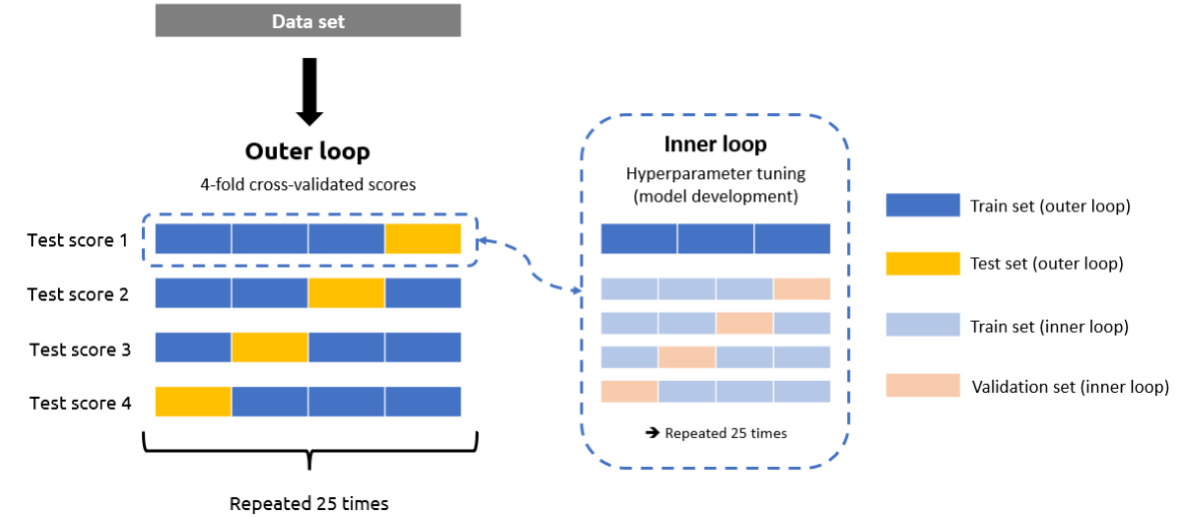
Illustration of the nested cross-validation method.

An alternative method called nested cross-validation is depicted in Figure 1. This method bypasses the limitations of the classical cross-validation approach [59]. In nested cross-validation, an “outer loop” cross-validation is applied to split the data set into several folds to compute the overall accuracy. In each run, an “inner loop” cross-validation is performed to tune and validate the model by means of the folds used to fit the model (train set) in the outer loop. When inner-loop cross-validation is performed, the model is refitted based on the best hyperparameters identified on the folds used as train sets, and its accuracy is obtained from the fold used as the test set. In this study, we used the nested cross-validation method with hyperparameter tuning, and we repeated the procedure 25 times to achieve the most robust results possible, following guidelines provided by Vabalas et al [59] and Krstajic et al [61]. To select our machine learning model, we followed the flowchart provided by Scikit-learn’s documentation and concluded that elastic net regression is suited to our aim, considering our sample size and the number of variables used (sample N<100,000, and few features are used). Thus, the linear regression model elastic net, which combines ridge and lasso penalties, was used for our analyses [62]. A seed value of 1 was set for replicable results. In the Results section, we report a mean *R*^2^ for each model computed as we obtained 1 *R*^2^ per run (4 × 25 runs were computed; see Figure 1). We then computed the adjusted *R*^2^ based on the formula 1 – [(N – 1)/(N – p – 1)] × (1 – *R*^2^), where p is the number of independent variables used in the model [63]. Finally, we compared the adjusted *R*^2^ of the models using an independent *t* test.

Traditional statistics (Mann-Whitney U test, Spearman rank correlations, Kruskal-Wallis test, Wilcoxon signed-rank test, and multiple linear regression) were computed using R version 4.0.3 (R Foundation for Statistical Computing), and machine learning analyses (elastic net regression) were computed using the Scikit-learn version 0.24 Python module [64]. As most study variables did not follow a normal distribution, preliminary analyses were conducted to support the use of a linear supervised machine learning–based elastic net regression. We thus computed 1 traditional multiple linear regression and 2 generalized linear models (negative binomial and quasi-Poisson regressions). These 3 models all presented a significant P value (<.001) and showed similar results. Additional preliminary analyses are available from the OSF [36]. Internal consistency (Cronbach α) for all questionnaires used in the study was computed using Spearman rank correlations.

## Results

### Objective 1: Comparison of Cyberchondria Scores Before and During COVID-19

As shown in Table 2, a series of Wilcoxon signed-rank tests showed significantly higher scores during the pandemic on 2 facets of the CSS-12 (*compulsion* and *distress* subscales) than before the pandemic. Table 2 also shows significantly lower scores on the *reassurance* subscale of the CSS-12 during the pandemic and no significant differences before and during the COVID-19 pandemic on the *excessiveness* subscale of the CSS-12 and the total CSS-12 score. Gender, age, and education effects on the CSS-12 scores during COVID-19 are reported in Table 3. There were no gender differences with regard to the CSS-12 subscales and total scores. Age and education had some effect on the CSS-12 subscales and total scores, as shown in Table 3.

**Table 3:** Gender, age, and education effects on the CSS-12^a^ scores during COVID-19.

Characteristics, tests, and groups		n (%)	CSS-12 total scores			CSS-12 excessiveness subscale scores			CSS-12 distress subscale scores			CSS-12 reassurance subscale scores			CSS-12 compulsion subscale scores	
			Median	Test result	Median		Test result	Median		Test result	Median		Test result	Median		Test result
**Gender, Mann-Whitney U test**																
	Female	416 (57.4)	26	*z*=–0.413, *P*=.68	9		*z*=–0.013, *P*=.99	7		*z*=–1.362, *P*=.17	5		*z*=–1.075, *P*=.28	4		*z*=–1.567, *P*=.12
	Male	302 (41.7)	26	*z*=–0.413, *P*=.68	9		*z*=–0.013, *P*=.99	6		*z*=–1.362, *P*=.17	5		*z*=–1.075, *P*=.28	4		*z*=–1.567, *P*=.12
**Age (years), Kruskal-Wallis H test**																
	15-24	248 (34.2)	28	*χ*²_4_=22.9, *P*<.001	10		*χ*²_4_=32, *P*<.001	7		*χ*²_4_=20.2, *P*<.001	5		*χ*²_4_=7, *P*=.14	4		*χ*²_4_=6.8, *P*=.15
	25-34	204 (28.1)	26	*χ*²_4_=22.9, *P*<.001	10		*χ*²_4_=32, *P*<.001	6.5		*χ*²_4_=20.2, *P*<.001	5		*χ*²_4_=7, *P*=.14	4		*χ*²_4_=6.8, *P*=.15
	35-44	117 (16.1)	26	*χ*²_4_=22.9, *P*<.001	9		*χ*²_4_=32, *P*<.001	6		*χ*²_4_=20.2, *P*<.001	5		*χ*²_4_=7, *P*=.14	4		*χ*²_4_=6.8, *P*=.15
	45-54	91 (12.6)	22	*χ*²_4_=22.9, *P*<.001	8		*χ*²_4_=32, *P*<.001	6		*χ*²_4_=20.2, *P*<.001	4		*χ*²_4_=7, *P*=.14	3		*χ*²_4_=6.8, *P*=.15
	≥55	65 (9.0)	25	*χ*²_4_=22.9, *P*<.001	9		*χ*²_4_=32, *P*<.001	6		*χ*²_4_=20.2, *P*<.001	5		*χ*²_4_=7, *P*=.14	3		*χ*²_4_=6.8, *P*=.15
**Education, Kruskal-Wallis H test**																
	Lower secondary	23 (3.2)	25	*χ*²_4_=10.8, *P*=.03	8		*χ*²_4_=11.8, *P*=.02	6		*χ*²_4_=15.1, *P*=.004	4		*χ*²_4_=12.4, *P*=.02	4		*χ*²_4_=2.6, *P*=.63
	Upper secondary	102 (14.1)	25	*χ*²_4_=10.8, *P*=.03	9		*χ*²_4_=11.8, *P*=.02	6		*χ*²_4_=15.1, *P*=.004	5		*χ*²_4_=12.4, *P*=.02	4		*χ*²_4_=2.6, *P*=.63
	Bachelor’s degree	308 (42.5)	26	*χ*²_4_=10.8, *P*=.03	9		*χ*²_4_=11.8, *P*=.02	7		*χ*²_4_=15.1, *P*=.004	5		*χ*²_4_=12.4, *P*=.02	4		*χ*²_4_=2.6, *P*=.63
	Master’s degree	236 (32.6)	26	*χ*²_4_=10.8, *P*=.03	10		*χ*²_4_=11.8, *P*=.02	7		*χ*²_4_=15.1, *P*=.004	5		*χ*²_4_=12.4, *P*=.02	4		*χ*²_4_=2.6, *P*=.63
	PhD	56 (7.7)	22	*χ*²_4_=10.8, *P*=.03	8		*χ*²_4_=11.8, *P*=.02	5		*χ*²_4_=15.1, *P*=.004	4		*χ*²_4_=12.4, *P*=.02	4		*χ*²_4_=2.6, *P*=.63

^a^CSS-12: Cyberchondria Severity Scale – Short Form.

### Objective 2: Psychological Factors Predicting Cyberchondria During COVID-19

The 3 facets of the CSS-12, which proved to be affected by the COVID-19 context (*distress*, *compulsion*, *reassurance*) were considered in relation to our second objective, which was to identify the best predictors of pandemic-related cyberchondria. To select the variables to be included in the computed supervised machine learning–based models, the correlations with the 3 retained CSS-12 subscales were considered (the entire correlation matrix is reported in Table 4). As no correlation reached the threshold of ρ≥0.30 [55,56] for the *reassurance* subscale, this facet was not considered in further analysis. In contrast, potential predictor variables were identified for the *distress* and *compulsion* subscales.

**Table 4:** Internal reliability coefficients (Cronbach α) and Spearman correlations between the variables.

	Cronbach α	1	2	3	4	5	6	7	8	9	10	11	12	13	14	15	16	17
1. CSS-12^a^: total score of cyberchondria	.89																	
2. CSS-12: excessiveness	.75	0.81^b^																
3. CSS-12: distress	.85	0.83^b^	0.55^b^															
4. CSS-12: reassurance	.78	0.73^b^	0.45^b^	0.49^b^														
5. CSS-12: compulsion	.82	0.77^b^	0.49^b^	0.56^b^	0.5^b^													
6. RQ^c,d^: secure attachment	N/A^e^	0.02	0.05	–0.02	0.03	0.02												
7. RQ: preoccupied attachment	N/A	0.16^b^	0.19^b^	0.18^b^	0.04	0.08^f^	–0.06											
8. RQ: fearful attachment	N/A	0.23^b^	0.18^b^	0.22^b^	0.17^b^	0.16^b^	0	0.24^b^										
9. RQ: avoidant attachment	N/A	0.04	0.08^f^	–0.02	0.04	0.04	0.05	0.22^b^	–0.02									
10. s-UPPS-P^g^: lack of premeditation	.82	–0.09^f^	–0.1^b^	–0.06	–0.04	–0.04	–0.03	–0.04	–0.02	0.01								
11. s-UPPS-P: lack of perseverance	.88	0.07	0.06	0.05	0.06	0.05	–0.05	0.04	0.07^f^	0.01	0.42^b^							
12. s-UPPS-P: sensation seeking	.83	0.07	0.08^f^	0.01	0.07	0.06	0.08^f^	–0.04	0.08^f^	0.06	0.14^b^	–0.01						
13. s-UPPS-P: global urgency	.82	0.11^b^	0.10^b^	0.10^b^	0.08^f^	0.07	0.02	0.09^f^	0.09^f^	0.04	0.36^b^	0.19^b^	0.18^b^					
14. Age	N/A	–0.17^b^	–0.19^b^	–0.17^b^	–0.07^f^	–0.08^f^	0.06	–0.15^b^	–0.24^b^	–0.06	–0.02	–0.18^b^	–0.17^b^	–0.08^f^				
15. PHQ-15^h^: somatic symptoms	.79	0.18^b^	0.15^b^	0.22^b^	0.08^f^	0.12^b^	–0.04	0.18^b^	0.11^b^	–0.01	–0.06	0.01	–0.04	0.04	0			
16. MAC-RF^i^: COVID-19–related fears	.79	0.44^b^	0.28^b^	0.52^b^	0.24^b^	0.35^b^	0.01	0.18^b^	0.16^b^	0	–0.10^b^	–0.02	–0.03	0.05	–0.06	0.31^b^		
17. SHAI^j^: health anxiety	.87	0.47^b^	0.36^b^	0.49^b^	0.27^b^	0.36^b^	–0.04	0.21^b^	0.23^b^	–0.03	–0.04	0.05	–0.07	0.04	–0.02	0.37^b^	0.48^b^	
18. IUS-12^k^: intolerance of uncertainty	.92	0.32^b^	0.31^b^	0.32^b^	0.17^b^	0.19^b^	–0.08^f^	0.38^b^	0.29^b^	0.09^f^	–0.18^b^	0.08^f^	–0.12^b^	0.1^f^	–0.2^b^	0.20^b^	0.41^b^	0.42^b^

^a^CSS-12: Cyberchondria Severity Scale – Short Form.

^b^Correlation is significant at the 0.01 level (2-tailed).

^c^RQ: Relationship Questionnaire.

^d^Internal reliability coefficients are based on Spearman correlations and not reported for the RQ, as each attachment dimension is defined by a unique item.

^e^N/A: not applicable.

^f^Correlation is significant at the 0.05 level (2-tailed).

^g^s-UPPS-P: Short UPPS-P Impulsive Behavior Scale.

^h^PHQ-15: 15-Item Patient Health Questionnaire.

^i^MAC-RF: Multidimensional Assessment of COVID-19–Related Fears.

^j^SHAI: Short Health Anxiety Inventory.

^k^IUS-12: Intolerance of Uncertainty Scale – Short Form.

A first supervised machine learning–based elastic net regression was computed for the *distress* subscale of the CSS-12. The following predictors were considered in the analysis: COVID-19–related fears (MAC-RF; ρ=0.515, *P*<.001), health anxiety (SHAI; ρ=0.491, *P*<.001), and intolerance of uncertainty (IUS-12; ρ=0.315, *P*<.001). As displayed in Table 5, the elastic net regression computed a mean *R*^2^ of 0.344 (SD 0.059), and we obtained an adjusted *R*^2^ mean of 0.333 (SD 0.06, 95% CI 0.321-0.345). The 2 most important predictors of the cyberchondria-related *distress* facet during the pandemic were COVID-19–related fears and health anxiety.

A second supervised machine learning–based elastic net regression was computed for the *compulsion* subscale of the CSS-12. The following predictors were considered in the analysis: COVID-19–related fears (MAC-RF; ρ=0.348, *P*<.001) and health anxiety (SHAI; ρ=0.355, *P*<.001). Both predictors included in the model (COVID-19–related fears and health anxiety) contributed similarly to the cyberchondria-related *compulsion* facet during the pandemic. As shown in Table 5, the elastic net regression computed a mean *R*^2^ of 0.152 (SD 0.046), and we obtained an adjusted *R*^2^ mean of 0.143 (SD 0.047, 95% CI 0.133-0.152), which is significantly lower than the one obtained for the model predicting the *distress* facet during COVID-19 (t_198_=24.954, *P*<.001, 95% CI 0.175-0.205). The *distress* model contained 3 predictors, whereas the *compulsion* model contained only 2 predictors, which at least partly explains the lower explained variance for *compulsion*. It is, however, worth noting that the reported adjusted *R*^2^ considered the number of predictors entered in the model.

**Table 5:** Repeated nested cross-validation using elastic net regression.

Dependent variable	*R*^2^, mean (SD)	Adjusted *R*^2^, mean (SD; 95% CI)	RMSE^a^, mean (SD)	MAE^b^, mean (SD)	COVID-19–related fears coefficient, mean (SD)	Health anxiety coefficient, mean (SD)	Intolerance of uncertainty coefficient, mean (SD)
CSS-12^c^ distress subscale	0.344 (0.059)	0.333 (0.06; 0.321-0.345)	2.512 (0.109)	2.003 (0.09)	1.018 (0.073)	0.938 (0.075)	0.158 (0.088)
CSS-12 compulsion subscale	0.152 (0.046)	0.143 (0.047; 0.133-0.152)	2.294 (0.14)	1.776 (0.092)	0.609 (0.054)	0.505 (0.055)	Variable not incorporated in the predictive model

^a^RMSE: root-mean-square error.

^b^MAE: mean-absolute error.

^c^CSS-12: Cyberchondria Severity Scale – Short Form.

## Discussion

### Principal Findings

This study aimed to determine whether the levels of cyberchondria changed during the COVID-19 pandemic and to identify the psychological predictors of cyberchondria during the pandemic. The results suggest that the facets of cyberchondria were affected during the COVID-19 pandemic following distinguishable patterns: although the levels of cyberchondria-related *distress* and *compulsion* increased, the levels of *reassurance* decreased. Using a supervised machine learning approach, we found that COVID-19–related fears (as assessed by the MAC-RF) and health anxiety (as assessed by the SHAI) were strong predictors of cyberchondria-related *distress* and *compulsion* during the pandemic.

An increase in the scores on the *distress* and *compulsion* subscales of the CSS-12 during the pandemic indicates higher levels of distress and greater interference with functioning, resulting from repeated online health searches. Scores on the *reassurance* subscale of the CSS-12 decreased during the pandemic, which suggests that online health searches were less likely to be conducted for the purpose of looking for medical professionals’ advice. This is possibly a consequence of either a sharply decreased availability of nonvital medical services during the first wave of the pandemic or the avoidance of medical facilities due to the fear of contracting COVID-19. Taken together, this pattern of results suggests that in the COVID-19 context, excessive online health searches do not provide reassurance, which may make these searches more distressing and cause impairment. Along the same lines, it is possible to speculate that the inability to obtain reassurance or necessary information via online health searches is also likely to increase the perception of threat and the accompanying fear of COVID-19, which may drive further searches.

These findings are in agreement with the theoretical model of cyberchondria during the COVID-19 pandemic [14]. Furthermore, they are in accordance with a suggestion that the “fear of not knowing” is a critical cognitive dimension of fear during the pandemic, which might increase distress and anxiety-related behaviors [12,13].

The scores on the *excessiveness* subscale of the CSS-12 did not show significant changes during the COVID-19 pandemic, which indicates that the general proneness to performing repeated online health searches does not necessarily change in the pandemic context. Likewise, total CSS-12 scores did not change during the pandemic, suggesting that the use of total CSS-12 scores in research may not reflect relevant or meaningful alterations in the patterns of problematic online health searches. This has implications for future research as the CSS is the most frequently used scale to assess cyberchondria [19,38], and studies conducted in the pandemic context have relied mainly on total scores either of the CSS-12 [29-31] or of the original CSS [65,66]. Therefore, it is advisable for future research on cyberchondria to always use scores on the CSS subscales in addition to total CSS scores. Furthermore, our findings raise concerns about the construct of cyberchondria, as assessed by various versions of the CSS, and support the notion that the issue of how best to assess cyberchondria needs to be revisited [38].

In view of our findings about the total CSS scores and scores on the specific CSS subscales, we specifically examined the predictors of the *distress* and *compulsion* facets of the construct of cyberchondria during the COVID-19 pandemic. The finding that COVID-19–related fears and health anxiety emerged as the strongest predictors of the *distress* and *compulsion* subscales of the CSS-12 supports the theoretical model of cyberchondria during the COVID-19 pandemic [14], as this model stipulates that the fear of COVID-19 is a key factor that drives online health searches in the pandemic context. A specific fear of COVID-19 and a more general propensity to be concerned about health and disease, as reflected in the construct of health anxiety, are likely to interact so that they mutually amplify one another. Our finding also confirms a significant relationship between health anxiety and cyberchondria that has been reported by numerous studies [20,21,37,67,68]. Moreover, other research has found a significant relationship between COVID-19–related fears and cyberchondria [27-30].

Other variables that were investigated in this study (somatic symptoms, intolerance of uncertainty, impulsivity traits, and attachment styles) did not emerge as strong predictors of either the *distress* or the *compulsion* facet of cyberchondria during the COVID-19 pandemic. Interestingly, the intolerance of uncertainty was a strong predictor only of the *distress* subscale of the CSS-12 but less so than COVID-19–related fears and health anxiety. Both previous research [30-32,67,69] and the theoretical model of cyberchondria during the COVID-19 pandemic [14] postulate a role for the intolerance of uncertainty in cyberchondria, but this role needs to be further investigated and better understood, alongside the impact of the fear of COVID-19 and health anxiety. With regard to impulsivity traits, their correlations with all subscales of the CSS-12 were the lowest, supporting the view that cyberchondria is better conceptualized as a behavior characterized by compulsivity or reassurance seeking [19,38] rather than impulsivity.

### Limitations

Our study comes with some specific limitations. First, we could have included additional predictor variables in our analyses. For example, maladaptive metacognitive beliefs have been associated with cyberchondria, both outside the COVID-19 context [67] and during the COVID-19 pandemic [29]. Yet, we selected our candidate predictor variables largely on the basis of the theoretical model of cyberchondria during the COVID-19 pandemic [14]. Other limitations include (1) our reliance on self-report instruments that may be affected by response biases (eg, social desirability, poor self-reflection abilities, and recall bias); (2) the cross-sectional nature of the study, which prevented us from investigating any causal relationships; (3) the self-selected nature of our sample, implying that it may not necessarily be representative of the general population (eg, our sample was mostly composed of highly educated individuals; see Table 1); and (4) the retrospective assessment of the prepandemic levels of cyberchondria.

### Conclusion

This study contributes to the literature on cyberchondria in general and cyberchondria in the context of the COVID-19 pandemic in several ways. First, the facets of cyberchondria that pertain to distress and interference with functioning as a result of problematic online health searches became more prominent during the COVID-19 pandemic and were strongly predicted by COVID-19–related fears and health anxiety, supporting the theoretical model of cyberchondria during the COVID-19 pandemic [14]. Second, this is the first study of cyberchondria to use a supervised machine learning approach. Third, we showed that both cyberchondria as a multidimensional construct and its assessment need to be reexamined.

This study also confirms that cyberchondria is a public health issue of particular relevance during health crises, such as pandemics [14,15]. In such a context, it is important to identify factors that foster cyberchondria, because targeting these factors will contribute to efforts to prevent cyberchondria and tailor interventions for individuals displaying problematic online health searches.

## Abbreviations

CSS-12: Cyberchondria Severity Scale – Short Form
IUS-12: Intolerance of Uncertainty Scale – Short Form
MAC-RF: Multidimensional Assessment of COVID-19–Related Fears
OSF: Open Science Framework
PHQ-15: 15-Item Patient Health Questionnaire
RQ: Relationship Questionnaire
SHAI: Short Health Anxiety Inventory
s-UPPS-P: Short UPPS-P Impulsive Behavior Scale

## Data Availability

The data sets generated and analyzed during the study are available in the Open Science Framework (OSF) repository [36].
